# Supplementary material for: Comparative Physiology and Transcriptome Analysis Provides Insights into the Regulatory Mechanism of Albinotic Bambusa oldhamii
Source: Plants (Basel). 2023 Dec 6;12(24):4090. doi: 10.3390/plants12244090 (PMC10747108; doi:10.3390/plants12244090)
Supplement: Supplementary file 1 [file plants-12-04090-s001.zip › plants-2639632-supplementary.pdf]

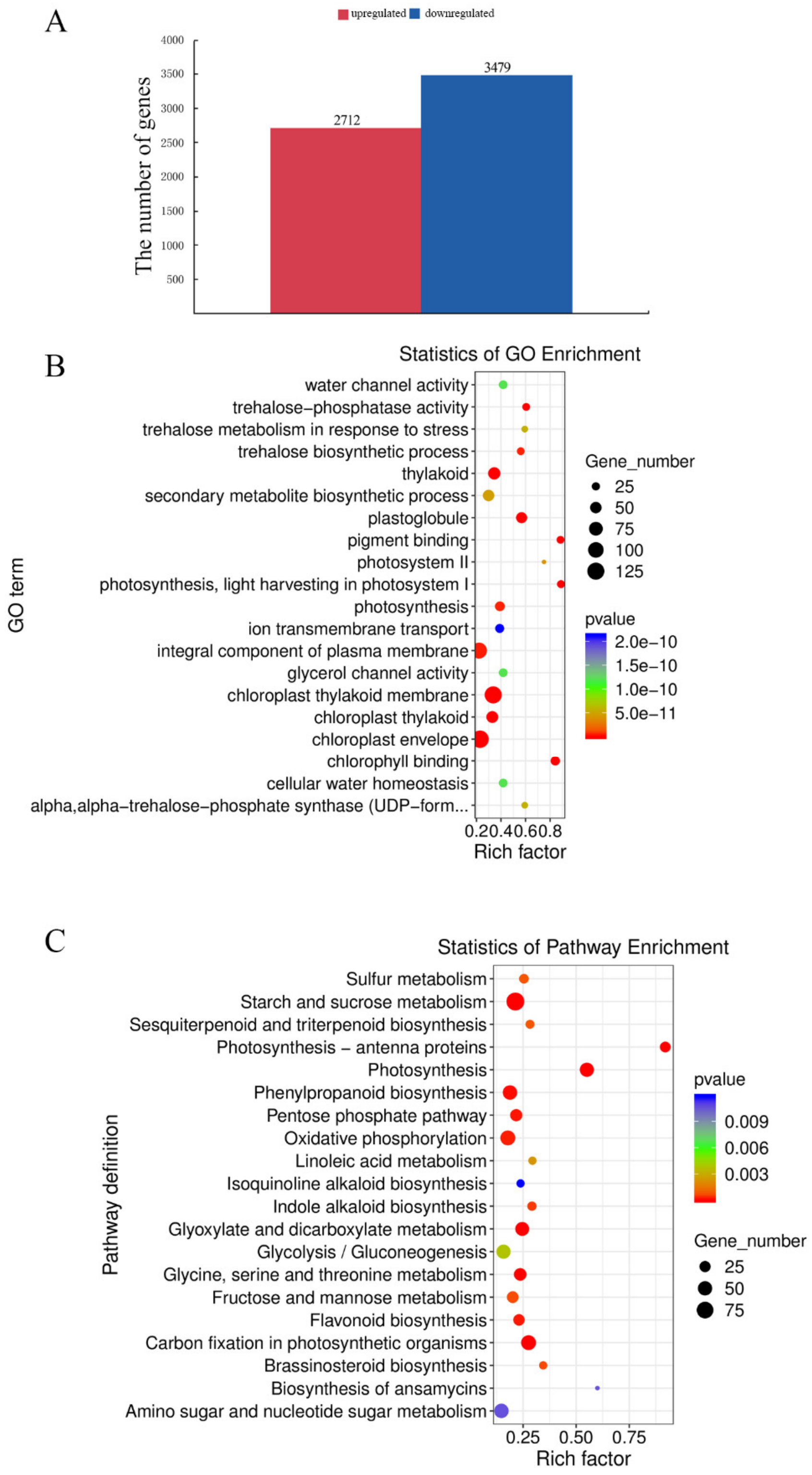

Figure S1. Statistical graph (A), GO analysis (B) and KEGG pathways (C) of differentially expressed genes in AL and NL

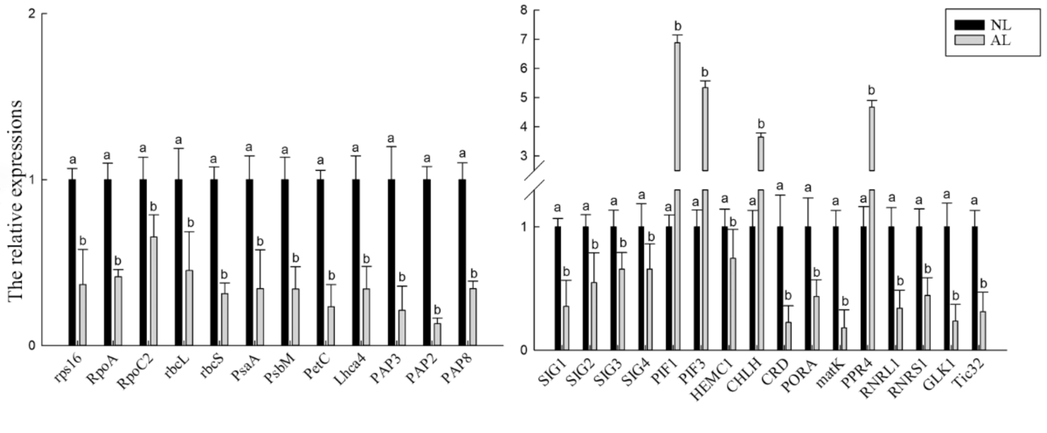

Figure S2. The verification of gene expressions by qRT-PCR

Table S1 Primary analysis of RNA-seq data

Samples	Raw reads	Clean reads	Proportion of clean reads	Q20%	Q30%	GC%
N1	39117414	38395976	98.16	98.36	95.02	51.71
N2	38934270	37900944	97.35	98.44	95.05	48.83
N3	41444372	39483684	95.27	98.32	94.75	49.27
A1	40755724	39884854	97.86	98.36	94.8	48.51
A2	37579836	36912228	98.22	98.31	94.67	48.41
A3	41293854	40416516	97.88	98.34	94.78	48.31

Note: N1,N2,N3: normal leaves; A1,A2,A3: albinotic leaves

Table S2 The primers used in qRT-PCR

Gene names	F	R
rps16	ACGTGCGACTTGAAGGACAT	TCTGTCCTCTCGAGCTCCAT
RpoA	TGGTTCGAGAGGAGGTAGCA	TGGCACGCTCCCAAATTTTG
RpoC2	GCAAAGATATCTCGGCGGGA	CGGCCCTCACAAATTGCAAA
rbcL	TCTTCACATTCACCGGGCAA	AGTCATTTCGCGTTCCCCTT
rbcS	TTCCCTGCCTCGAGTTTGAC	CTTGTGGACGACGAAGGACA
PsaA	GAAGCACATACTCCTCCGGG	AGCAAAAGCCCCTGTCATGA
PsbM	AGGCCCCTTCCATTTTGTAGT	GTGAGATGGAGAAAAGAGGGCT
PetC	TGGTGTGTACAATGGCAGCT	CGAGCAGGAGGAGGTTCATC

Lhca4	TGTTGGCGTTCCTAGGGTTC	TCCCCTGTACATCCTCTGCA
PAP3	CACTAGTGGAGGCGGTTCAA	CAGTGACATAGGTGGAGGGC
PAP2	TGTCCACAGGTACGAGAGGT	CAGGCTGTGGAAAGATGGGA
PAP8	CTGACCGGGGAGTGTTACTG	GGCTTCCACCACCTGAATCA
SIG1	GAGCAGGAACCGGATCTACC	AGCTGCTCGTATGATGGCTC
SIG2	GCCTCCCAAAGTACAGCGTA	TCCATCCCGAACCTGTACCT
SIG3	AGGGTGCAAATTCTCGACGT	TCGACGAGAGCACTTCCATG
SIG4	TTCGACGATATCTGCCAGGC	GTGGATCTCTTGCCTCTCCG
PIF1	GCCGTCACAGCTGTTTGAAA	TGCTGCCGAAACATCTCAGA
PIF3	GAGCGGAGAAGAAGGGATCG	ACATGCGGAGAAATGGGGAA
HEMC1	GCACACTCGGAGTTAGCTGA	TCGAGTGAACCGCAATGTCA
CHLH	TCATCGGCTCGCTCATCTTC	CGGAGTTCTTCTTGCGCTTG
CRD	TGCAGGAGTTCAAGACGGAC	CGGGTTGGTTTTCTTGAGCC
PORA	GGAGGGCAGCTACACCATTA	GTTCACTCCGACGCTCATCT
matK	AGACGCGAAAGTACAAGCCT	GATCGCCGCAGAAGTAGGAA
PPR4	AGCCTTGTTCACGCTTATGC	TGCCGATCTTGGTCTTAGCC
RNRL1	GATTCACCAGAGGCTCAGCA	TGTCAGGTTGGAGAATGCCC
RNRS1	ACTTCTGACGAACTGCCCTG	TCTGTGCATTGCTTCCCTGT
GLK1	GAGTTGTGCCGTGTCCAATG	CCGCAAGCTAGAGGGACATT
Tic32	TCTTGAGGAGCTTGGATGCC	GCTAATGTGACGGCCAACTG
NTB	TCTTGTTTGACACCGAAGAGGAG	AATAGCTGTCCCTGGAGGAGTTT
